# Indoor air bacterial load and antibiotic susceptibility pattern of isolates at Adare General Hospital in Hawassa, Ethiopia

**DOI:** 10.3389/fpubh.2023.1194850

**Published:** 2023-11-02

**Authors:** Yibeltal Assefa Atalay, Embialle Mengistie, Alemu Tolcha, Belete Birhan, Getachew Asmare, Natnael Atnafu Gebeyehu, Kelemu Abebe Gelaw

**Affiliations:** ^1^School of Public Health, College of Health Sciences, and Medicine, Wolaita Sodo University, Wolaita Sodo, Ethiopia; ^2^Department of Environmental Health, College of Health Sciences, and Medicine, Hawassa University, Hawassa, Ethiopia; ^3^Department of Psychiatry, College of Health Sciences, and Medicine, Wolaita Sodo University, Wolaita Sodo, Ethiopia; ^4^School of Midwifery, College of Health Sciences, and Medicine, Wolaita Sodo University, Wolaita Sodo, Ethiopia

**Keywords:** bacterial load, bacterial isolate, drug resistance, indoor air quality, Ethiopia

## Abstract

**Background:**

Air is the agent of pathogenic microbes that cause significant problems in the hospital environment. Multidrug resistance poses a major therapeutic challenge to these airborne microorganisms in hospital indoor environments.

**Method and materials:**

This study was conducted at Adare General Hospital in Hawassa City, Sidama, Ethiopia. A cross-sectional study was conducted. The proportional allocation method was used to select the sampled 50 rooms from the total available rooms in each category of wards and staff offices. A total of 100 indoor air samples were collected using settle plates in all units twice a day, morning (9:00–4:00 a.m.) and afternoon (3:00–4:00 p.m.). The types and number of colonies were determined in the laboratory, and the pathogenic bacteria were isolated by appropriate bacteriological techniques. Antimicrobial susceptibility testing was performed on Mueller-Hinton agar for each potentially pathogenic bacterium isolated. For each bacterium, a total of 12 antibiotics were tested using the Kirby-Bauer disk diffusion method. The test organism was adjusted to McFarland turbidity standards of 0.5. Data were entered and analyzed using the SPSS version 25 window. Descriptive analysis and one-way analysis of variance were performed.

**Results:**

The indoor air bacterial load of Adare General Hospital was found in the range between 210 and 3,224 CFU/m^3^. The highest indoor air bacterial load was identified from the gynecology ward with a mean of 2,542.5CFU/m^3^ at *p* < 0.05. From 100 indoor air samples, a total of 116 bacterial pathogen isolates were obtained. Gram-positive isolates predominated at 72.4%, of which 37.1% were *Staphylococcus aureus*, 26.7% were *coagulase-negative Staphylococci*, and the rest 8.6% were *Streptococcus pyogenes*. The isolation of pathogenic bacteria *Staphylococcus aureus* and *coagulase-negative Staphylococci* showed a high level of resistance to ampicillin.

**Conclusion:**

A high bacterial load was found in the study area as compared to different indoor air biological standards. *Staphylococcus aureus* and *coagulase-negative Staphylococci* were the isolated predominant bacteria. Attention should be given to preventing and minimizing those environmental factors that favor the multiplication of bacteria in the indoor environment of a hospital for the safe health of patients, visitors, and staff.

## Introduction

Hospital-acquired infections (HAI) are infections acquired in the healthcare service unit that appear 48 h or more after hospital admission or within 3 days after discharge up to 30 days after an operation in a healthcare facility when someone was admitted for reasons other than the infection (1). In hospital environments, the air is a carrier for pathogens that can cause hospital-acquired infection (2). Approximately 5–10% of patients admitted to modern hospitals in Western countries acquire one or more nosocomial infections. They are also associated with significant morbidity, mortality, and hospital costs (1, 3).

People inhale significant amounts of microorganisms called bioaerosols, and it contributes to ~5–34% of indoor air pollution in healthcare environments because they contain a diverse population of microorganisms (4, 5). Microorganisms, such as bacteria, fungi, and viruses, cause hospital-acquired infections. However, more than 90% of hospital-acquired infections are due to bacteria (5, 6).

The most common gram-positive bacterial pathogens causing nosocomial infections reported are *Staphylococcus aureus (S. aureus)* and *coagulase-negative Staphylococcus (CoNS)*, which may be coming from the patients, health personnel, attendants, contaminated instruments, and the environment (7).

Many strains of bacteria in health service environments are multidrug resistant. The widespread use of drugs, especially over or inappropriate use of antibiotics, contributed to an increased incidence of antimicrobial-resistant organisms (8, 9).

Nosocomial infections cause substantial morbidity, mortality, and economic loss. It can prolong hospital stays and intensive diagnostic and therapeutic procedures. Furthermore, the more recent challenges of nosocomial infections play an important role in increasing the emergency of multidrug-resistant microorganisms by collecting resistance-mediating genes (1, 10).

In 2018, 3.8 million deaths were attributed to indoor air pollution worldwide. In low- and middle-income countries, more than 90% of indoor air pollution-related deaths occur, mainly in Africa, and the Eastern Mediterranean region (11).

More than two million patients acquire infections attributed to air pollution per year in US hospitals, while they are hospitalized for other health problems. Due to airborne infections, 90,000 people die and 5–10 billion dollars is being imposed on the economy of this country each year (12).

The prevalence of nosocomial infection ranges from 2.5 to 14.8% in sub-Saharan Africa, with the cumulative incidence in surgical wards being very high due to influencing factors of a poor ventilation system, cleanness of the hospital wall, roof of rooms, overcrowding setting, coughing, and high movement of personnel in a hospital environment (13). Approximately 10% of nosocomial infections in both immune-suppressed and immune-competent people are caused by airborne bacteria (9).

Various studies in Ethiopia also showed that nosocomial infections are a significant problem that needs attention and action because isolated pathogenic bacteria are highly resistant to drugs in hospitals (10).

According to the World Health Organization (WHO) report, antimicrobial resistance is a challenge to global public health today. It has been detected in all parts of the world and the problem is increasing; its development and spread are being accelerated by the misuse of antimicrobial medicines, inadequate infection prevention, control programs, and insufficient regulation for the use of antimicrobial medicines (14).

Based on reports from kinds of literature, various studies in different hospitals in Ethiopia focused on only inpatient wards and showed that the indoor air bacterial load of the hospital was beyond expected standards and isolated organisms are highly resistant to commonly prescribed drugs.

However, in the present study area, there is quite limited data on the indoor air bacterial load, isolates, and antibiotic susceptibility tests from different outpatient, inpatient departments, and staff offices. This study was conducted to fill these gaps in indoor air bacterial load difference between staff offices, outpatient, and inpatient departments, and it is very crucial to assess influencing factors for bacterial load in each department of the hospital. Therefore, the main aim of this study was to assess the indoor air bacterial load, isolates, and antimicrobial susceptibility patterns at Adare General Hospital. It provided important information on the quality of indoor air for hospital managers. This study was given baseline information for implementing evidence-based strategies for infection prevention and control measures at healthcare facilities.

## Methods and materials

### Study area and period

The study was conducted at Adare General Hospital (AGH) in Hawassa City. The hospital has a capacity of 320 beds, a total of 444 healthcare professionals, and 125 support staff. The hospital also offers daily services to approximately 400 clients. It provides services to ~6,000 inpatients and 70,000 outpatients a year from the population served.

The hospital offers services such as outpatients, emergency rooms, hospitalized patients (internal medicine, pediatrics, neonatal intensive care, delivery, and surgery), laboratory, radiology, and dental clinic. It also serves as a training center for health science students from government and private educational institutions.

A laboratory-based cross-sectional study design was used to assess indoor air bacterial load isolates and the antimicrobial susceptibility patterns at Adare General Hospital wards and offices from July to November 2021.

### Sites and sample collection

The raw data were obtained from the laboratory investigation and the observation checklist.

Samples were collected from the following wards: Pediatrics Ward (PW), Surgical Ward (SW), Medical Ward (MW), Gynecology Ward (GW), Optometry Ward (OW), staff offices, and Emergency Outpatient Department (EOPD). A total of 50 plate media were used to collect air samples twice daily that contained blood agar for the general growth of bacteria, mannitol salt agar, and MacConkey agar for the selective growth of bacteria.

Air samples were collected twice daily, during the morning (9:00–10:00 a.m.) and afternoon (3:00–4:00 p.m.). Currently, the high density of occupants, the activities of staff in offices, and the number of visitors are taken into consideration. The ambient temperature was measured by the temperature meter. Samples were collected as standard protocol using the settle plate method following a 1/1/1 schedule to measure bacterial load using 90-mm-diameter sterile Petri dishes containing 5% sheep's blood agar left open to the air for an hour (15). During the air sampling procedure, sterile gloves and protective gowns were worn to prevent self-contamination of the agar plates. The plates were labeled with the sample number, room type, parts, and the date and time the samples were taken. After collection, the plates were covered with their lids and transported in a cold box to the Hawassa University Comprehensive Specialized Hospital (HU-CSH) microbiology laboratory for microbiological testing.

### Sampling process and laboratory analysis

The inoculated blood agar plates were incubated at 35–37°C for 24–48 h. The culture plates that show distinct colonies were counted using the plate colony counter. The microbial concentration of air was expressed in colony-forming units (16). Then, colonies were examined for the growth of potentially pathogenic bacteria initially by colony characteristics, hemolysis, and microscopic examination of Gram-stained smears. Then, these suspected colonies were sub-cultured on mannitol salt agar (MSA) and MacConkey was used accordingly, and finally, all positive cultures on blood agar with significant bacteria were identified at the species level by their colony characteristics, gram-staining reaction, and the pattern of biochemical profile by using standard procedures.

Gram-negative bacteria were identified by citrate utilization, motility test, urease test, and oxidase and carbohydrate utilization tests. The gram-positive bacteria were identified by using catalase and coagulase tests. Antimicrobial susceptibility tests of all isolated bacteria from air samples were performed according to the Laboratory Standards Institute method (17, 18). The antimicrobial susceptibility pattern of isolated bacteria was determined based on the disk diffusion technique using Mueller–Hinton agar (Oxoid, UK) for potential pathogenic bacteria isolated with 12 antibiotics. The suspension of the test organism was prepared by picking parts of similar test colonies with a sterile wire loop, suspended in sterile normal saline, and incubated for 2 h to allow organisms to reach their log phase in growth. The densities of suspension or turbidity of bacteria to inoculate were determined by comparison with the standard on McFarland 0.5 Barium sulfate solution (19).

### Data quality and management

The reliability of the study findings was guaranteed by implementing recommended quality control measures throughout the whole process of the laboratory work. During the laboratory work, all laboratory materials were sterilized. Aseptic techniques were used in all steps of specimen collection, transportation, and inoculation onto culture media to minimize contamination. All culture plates were prepared according to the manufacturer's instructions.

In addition, to assure the quality of data the expired date of all culture plates, reagents and drugs were checked. Culture media were tested for sterility and performance and checked daily to observe whether cracks and contamination formed during culture processes (17).

### Data interpretation and statistical analysis

The data entry, cleaning, and analysis were performed using SPSS version 26 software. First, descriptive statistics were done using frequency and percentage to present the generated data in the form of graphs and tables. One-way analysis of variance was used to assess the mean indoor air bacterial load difference among different wards and staff offices. After 24-h incubation period, the bacterial colony was counted by colony counter, and bacterial load was expressed as colony-forming units and CFU/m^3^ by using the following formula, which is described by Omeliansky: *N* = 5a.10^4^(b. t)^−1^, where *N* = microbial CFU/m^3^ of indoor air (cubic meter air), a = number of colonies per plates, b = dish surface area (63.585 cm^2^), t = exposure time in minutes, 10^4^ = factors (20).

## Results

### The indoor air bacterial load

The result of this study indicated that the highest bacterial load was found in the gynecology and pediatric wards, which was 3,224 CFU/m^3^, and the lowest bacterial concentration was recorded in the staff office, which was 210 CFU/m^3^ during 60 m exposure. Then, indoor air bacterial load of AGH was found in the range between 210 and 3,224 CFU/m^3^ (Table 1). Based on the standards set by WHO, the indoor air bacterial loads were grouped into <1,000 CFU/m^3^ (acceptable) and >1,000 CFU/m^3^ (unacceptable) in the hospital. Based on these, the chi-square results of this study regarding the time of sample collection, the morning sample of bacterial load in the air were slightly unacceptable 45/50 (90.0%) than the afternoon samples 42/50 (84.0%), with a *p*-value of 0.372 (Table 2).

**Table 1:** One-way analysis of variance for mean indoor air bacterial load of wards and staff offices at AGH, in Hawassa City, 2021.

**CFUm^3^**	***N***	**Mean CFU/m^3^**	**Std. deviation**	**Std. error**	**95% CI for mean**		**Minimum**	**Maximum**
					**Lower**	**Upper**		
Surgical ward	12	2,166.83	746.944	215.624	1,692.24	2,641.41	813	2,988
Medical ward	16	2,132.97	611.870	152.967	1,806.93	2,459.01	891	3,041
Gynecology ward	14	2,542.53	540.807	144.537	2,230.28	2,854.78	1,730	3,224
Pediatric ward/NICU	12	2,535.98	452.500	130.626	2,248.47	2,823.48	1,809	3,224
Optometry	6	2,035.77	593.256	242.196	1,413.19	2,658.35	1,337	2,883
Staff office	30	1,355.14	623.676	113.867	1,122.26	1,588.03	210	2,438
Emergency OPD	10	1,536.00	459.381	145.269	1,207.38	1,864.62	944	2,438
Total	100	1,943.85	747.063	74.706	1,795.62	2,092.09	210	3,224

**Table 2:** The time variation of bacterial load against the standards at AGH, in Hawassa City, 2021.

**Acceptability of bacterial loads**	**Time [number of samples** ***N*** = **50 (%)]**			***X*^2^ (*P*-value)**
	**Morning**	**Afternoon**	**Total (%)**	
Acceptable	5 (10.0)	8 (16.0)	13 (13.0)	0.372
Unacceptable	45 (90.0)	42 (84.0)	87 (87.0)	
Total	50	50	100	

**Figure 1:**
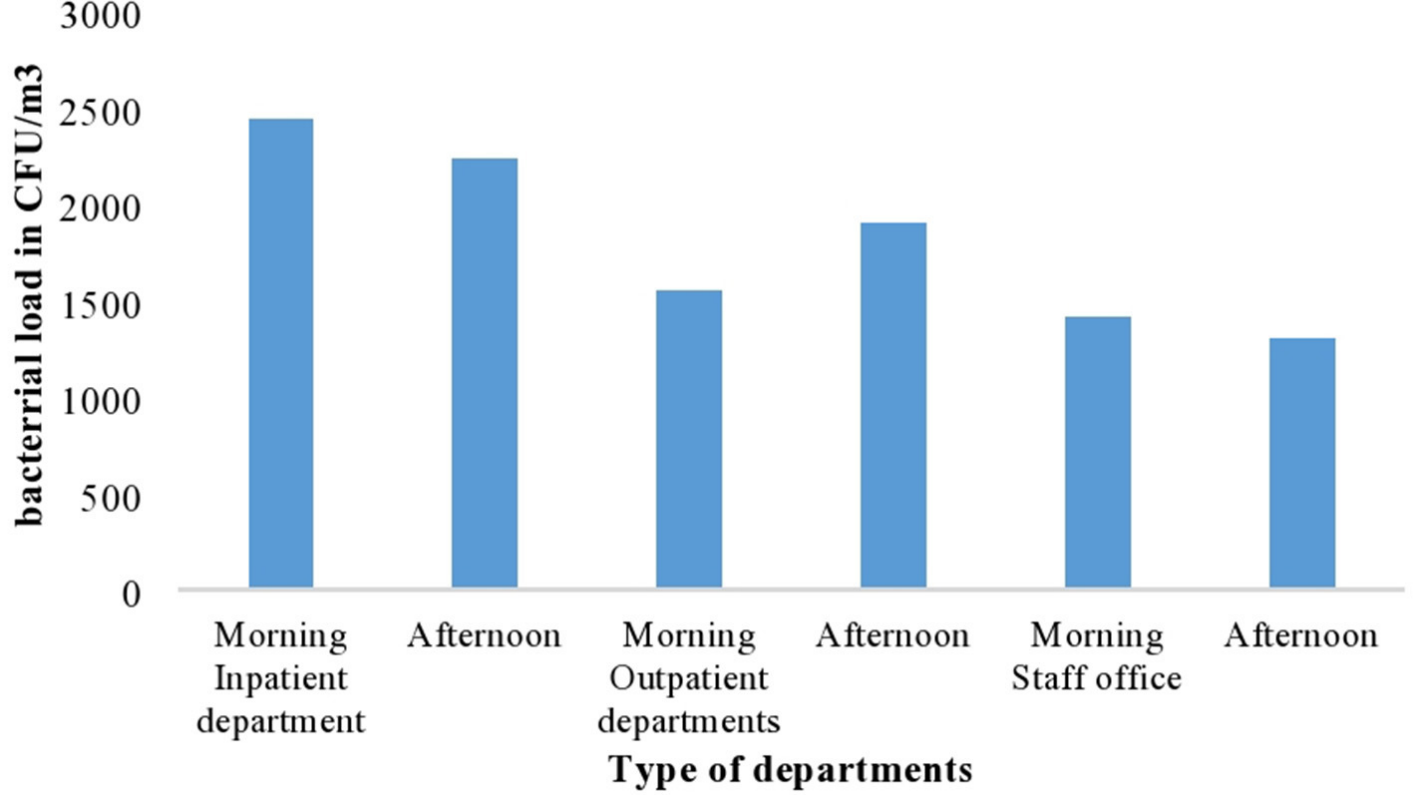
The mean indoor air bacterial load at different departments with different period of sample collection at AGH in Hawassa City, 2021.

According to this study, a significant difference in mean bacterial load was found among the three departments of the hospital (*p* < 0.001). The highest mean bacterial load was found in the inpatient department of the hospital (2,335.93 CFU/m^3^) and the lowest mean bacterial load was registered in the staff office (1,354.96 CFU/m^3^) (Figure 1).

The researcher also determined the degree of indoor air bacterial load from different wards and offices based on a collected sample. In terms of the distribution of wards and offices, the one-way analysis of variance test was conducted to compare the average bacterial load of wards as presented below. The highest mean indoor air bacterial load was identified from gynecology wards with a mean of 2,542.5 CFU/m^3^ at a *p*-value of <0.001^*^, followed by pediatric ward/NICU with a mean of 2,536.0 CFU/m^3^ at a *p*-value of <0.001^*^ and surgical ward with mean of 2,166.8 CFU/m^3^ at a *p*-value of =0.003.

However, the lowest indoor air bacterial loads were detected at the staff office with a mean of 1,355 followed by the optometry ward with a mean of 1,536.0 CFU/m^3^ at a *p*-value of <0.001. The total average concentration of bacterial concentration was 1,943.85 CFU/m^3^ in both morning and afternoon (Table 1). ANOVA test result was presented to show the mean bacterial concentration difference among different wards. The test showed that there was a significant mean bacterial concentration difference among wards at a *p*-value of <0.001 (Table 3).

**Table 3:** ANOVA test results on bacterial concentration difference among different wards and staff offices at AGH in Hawassa City, 2021.

**CFUm^3^**	**Sum of squares**	**Df**	**Mean square**	***F***	**Sig**.
Between groups	22,505,508.062	6	3,750,918.010	10.653	0.000
Within groups	32,746,643.259	93	352,114.444		
Total	55,252,151.321	99			

### Dominant types of isolated bacteria

**Figure 2:**
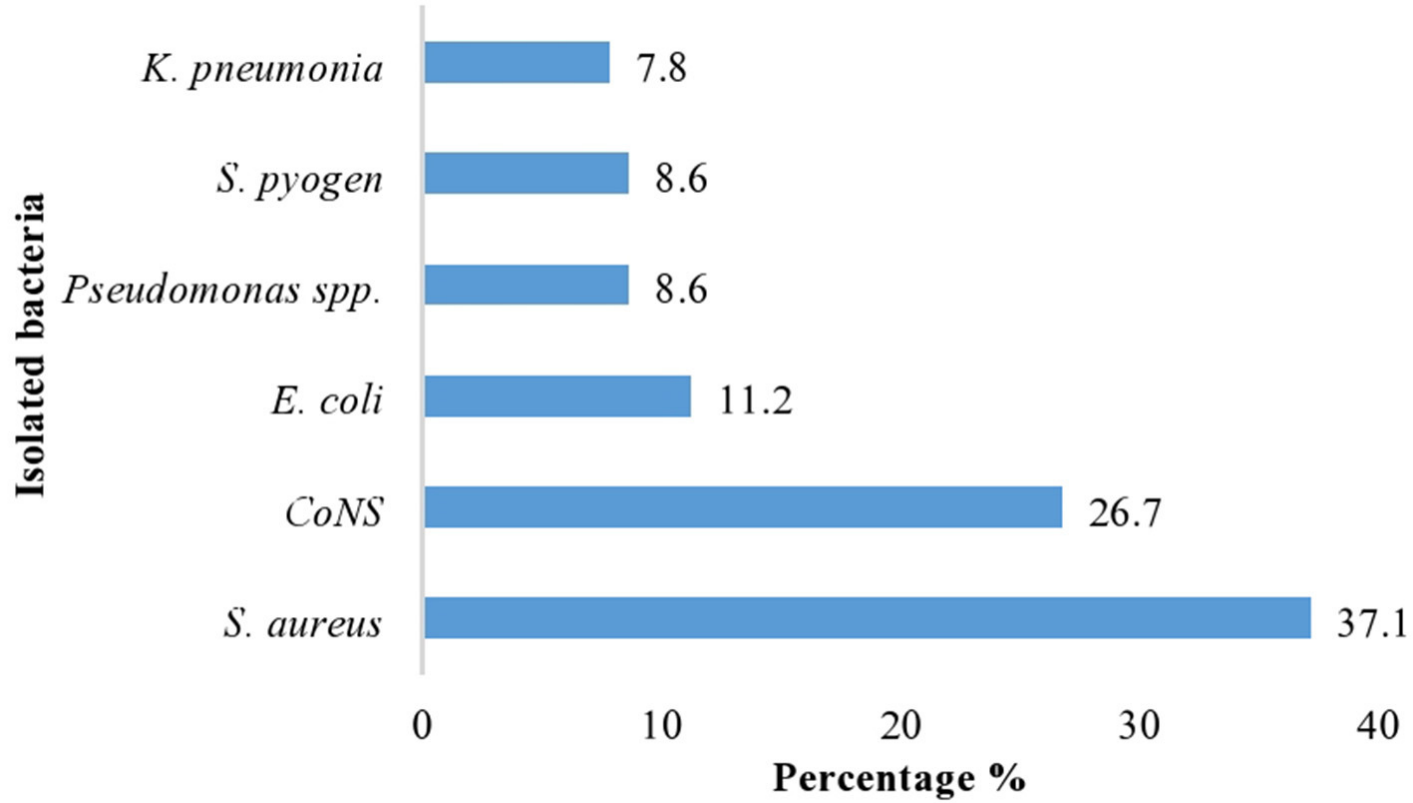
Dominant types of bacterial isolates at Adare General Hospital in Hawassa City, 2021.

A total of 100 air samples were collected in the morning and afternoon from seven different wards. From 100 indoor air samples, a total of 116 bacterial pathogens were isolated. Among these, gram-positive isolates bacteria were 84 (72.4%), of which *S*. *aureus* were 43 (37.1%), *coagulase-negative Staphylococci* bacteria were 31 (26.7%), and the rest *S. pyogens* were 10 (8.6%). Similarly, while gram-negative isolates were 32 (27.6%), among which *E. coli* were 13 (11.2%), pseudomonas species were 10 (8.6%), and *K. pneumonia* were 9 (7.8%), which were the main identified pathogenic bacteria (Figure 2).

### Antimicrobial susceptibility patterns

The susceptibility patterns of pathogenic bacteria isolated with 12 selected antibiotics were determined by the disk diffusion technique. Gram-positive isolate test showed that 60.5% of the *S. aureus* showed high-level resistance to ampicillin, whereas 55.8, 41.9, 39.5, and 34.9% of the *S. aureus* showed resistance to chloramphenicol, clindamycin, ciprofloxacin, and trimethoprim/sulfamethoxazole (SXT), respectively. Similarly, 86.0% of *S. aureus* showed susceptibility to penicillin and 83.7% to both ceftriaxone and gentamicin.

The *coagulase-negative Staphylococci* showed that 48.4% of the *CoNS* bacteria were resistant to both tetracycline and ciprofloxacin, and 45.2% of the *coagulase-negative Staphylococci* were resistant to both vancomycin and ceftriaxone. Similarly, while 96.8, 87.1, 83.9, and 71.0% of the *coagulase-negative Staphylococci* have susceptibility to gentamicin, chloramphenicol, ampicillin, and cephalexin, respectively, the other gram-positive bacteria test showed that 70, 60, and 50% of the *S. pyogene* have high-level resistance to ceftriaxone, erythromycin, and vancomycin, respectively.

Regarding the gram-negative bacteria, a test showed that 60% of the Pseudomonas spp. were resistant to cephalexin, whereas 50% of Pseudomonas spp. were resistant to both ciprofloxacin and gentamicin. Similarly, 100.0% of the Pseudomonas spp. bacteria were susceptible to ampicillin and 90.0% to chloramphenicol. The other gram-negative isolates bacteria test showed that 46.2% of *E. coli* were also resistant to ciprofloxacin. Similarly, 100.0% of the *E. coli* bacteria were susceptible to gentamicin. In addition to this *K. pneumonia* bacteria, a test showed 77.8% resistance to cephalexin. Similarly, 100.0% of *K. pneumonia* bacteria were susceptible to chloramphenicol, gentamicin, and tetracycline (Table 4).

**Table 4:** Antimicrobial susceptibility patterns of isolated pathogenic bacteria at AGH in Hawassa City, 2021.

**Antibiotics**		**Isolated bacteria**											
		***S. aureus***		***CoNS***		**Pseudomonas spp**.		***S. pyogenic***		***E. coli***		***K. pneumonia***	
		**No**.	**(%)**	**No**.	**(%)**	**No**.	**(%)**	**No**.	**(%)**	**No**.	**(%)**	**No**.	**(%)**
Ampicillin	S	17	(39.5)	26	(83.9)	10	(100.0)	7	(70.0)	9	(69.2)	4	(44.4)
	R	26	(60.5)	5	(16.1)	0	(0.0)	3	(30.0)	4	(30.8)	5	(55.6)
Ceftriaxone	S	36	(83.7)	17	(54.8)		NA	3	(30.0)	9	(69.2)	4	(44.4)
	R	7	(16.3)	14	(45.2)		NA	7	(70.0)	4	(30.8)	5	(55.6)
Chloramphenicol	S	19	(44.2)	27	(87.1)	9	(90.0)	6	(60.0)	9	(69.2)	9	(100.0)
	R	24	(55.8)	4	(12.9)	1	(10.0)	4	(40.0)	4	(30.8)	0	(.0)
Ciprofloxacin	S	26	(60.5)	16	(51.6)	5	(50.0)	6	(60.0)	7	(53.8)	4	(44.4)
	R	17	(39.5)	15	(48.4)	5	(50.0)	4	(40.0)	6	(46.2)	5	(55.6)
Clindamycin	S	25	(58.1)	20	(64.5)		NA	5	(50.0)		NA		NA
	R	18	(41.9)	11	(35.5)		NA	5	(50.0)		NA		NA
Erythromycin	S	36	(83.7)	18	(58.1)		NA	4	(40.0)		NA		NA
	R	7	(16.3)	13	(41.9)		NA	6	(60.0)		NA		NA
Gentamicin	S	36	(83.7)	30	(96.8)	5	(50.0)	10	(100.0)	13	(100.0)	9	(100.0)
	R	7	(16.3)	1	(3.2)	5	(50.0)	0	(0.0)	0	(0.0)	0	(0.0)
Penicillin	S	37	(86.0)	19	(61.3)		NA	6	(60.0)	8	(61.5)	7	(77.8)
	R	6	(14.0)	12	(38.7)		NA	4	(40.0)	5	(38.5)	2	(22.2)
SXT	S	28	(65.1)	18	(58.1)		NA	10	(100.0)		NA		NA
	R	15	(34.9)	13	(41.9)		NA	0	(0.0)		NA		NA
Cephalexin	S	35	(81.4)	22	(71.0)	4	(40.0)	6	(60.0)	9	(69.2)	2	(22.2)
	R	8	(18.6)	9	(29.0)	6	(60.0)	4	(40.0)	4	(30.8)	7	(77.8)
Tetracycline	S	26	(60.5)	16	(51.6)		NA	6	(60.0)	9	(69.2)	9	(100.0)
	R	17	(39.5)	15	(48.4)		NA	4	(40.0)	4	(30.8)	0	(0.0)
Vancomycin	S	35	(81.4)	17	(54.8)		NA	5	(50.0)		NA		NA
	R	8	(18.6)	14	(45.2)		NA	5	(50.0)		NA		NA

S, sensitive/susceptible; R, resistant; NA, not applicable.

The overall multidrug resistance (MDR) patterns of isolated bacteria showed that 76 (90.5%) of total isolated gram-positive bacteria were resistant to more than two antibiotics tested. Similarly, of the isolated gram-negative bacteria, only 25 (78%) were found resistant to more than two antimicrobial classes. Approximately 87% of the 116 (both gram-positive and negative) pathogens isolated were found resistant to more than two of the antibiotics tested (Table 5).

**Table 5:** Multi-drug resistance patterns of isolated bacteria at AGH, in Hawassa City, 2021.

	**MDR patterns**											
	**R0**		**R1**		**R2**		**R3**		**R4**		≥**R5**	
**Isolated bacteria**	**No**.	**(%)**	**No**.	**(%)**	**No**.	**(%)**	**No**.	**(%)**	**No**.	**(%)**	**No**.	**(%)**
Gram negative	0	(0.0)	7	(21.9)	13	(40.6)	10	(31.3)	1	(3.1)	1	(3.1)
Gram positive	4	(4.8)	4	(4.8)	12	(14.3)	14	(16.7)	22	(26.2)	28	(33.3)
*S. aureus*	2	(4.7)	3	(7.0)	8	(18.6)	8	(18.6)	13	(30.2)	9	(20.9)
*CoNS*	2	(6.5)	0	(0.0)	4	(12.9)	4	(12.9)	8	(25.8)	13	(41.9)
*Pseudomonas* spp.	0	(0.0)	5	(50.0)	3	(30.0)	2	(20.0)	0	(0.0)	0	(0.0)
*S. pyogenic*	0	(0.0)	1	(10.0)	0	(0.0)	2	(20.0)	1	(10.0)	6	(60.0)
*E. coli*	0	(0.0)	1	(7.7)	8	(61.5)	3	(23.1)	0	(0.0)	1	(7.7)
*K. pneumonia*	0	(0.0)	1	(11.1)	2	(22.2)	5	(55.6)	1	(11.1)	0	(0.0)

R0, sensitive to all drugs; R1, Resistance to one drug; R2, Resistance to two drugs; R3, resistance to three drugs; R4, Resistance to four drugs; R5, Resistance to five drugs; No., number of pathogenic bacteria isolated.

### Contributing factors for indoor air bacterial load

Environmental factors were tested for an association with indoor bacterial loads. The result of this study revealed a negative significant correlation (*P* < 0.001) of total bacterial load with cleaning frequency (*r* = −0.634) and temperature (*r* = −0.559). Human factors were also tested for the correlation with indoor air bacterial loads. The result of this study also revealed a positive significance (*p* < 0.001) of the total indoor air bacterial load with the number of people present in the rooms during sampling (*r* = 0.726).

A multiple linear regression model predicting total indoor air bacterial load in the hospital was developed. A significant association was found between total bacterial load and explanatory variables such as room temperature, frequency of cleaning, proper use of ventilation system of rooms, cleanness of the ceiling of the room, and the number of people present in the room. However, there was no significant association between indoor air bacterial load, the cleanness of the wall, and the cleanness of the floor of the rooms (*P* > 0.05) (Table 6).

**Table 6:** Multiple linear regression analysis models using the indoor bacterial load as a continuous dependent variable.

**Predictor variables**	**Unstandardized coefficients**		**Standardized coefficient**			**95.0% confidence interval for B**	
						**Lower bound**	**Upper bound**
	***B***	**Std. error**	***Beta***	***T***	**Sig**.		
The temperature of the rooms	−2.922	1.145	−0.136	−2.551	0.012	−5.197	−0.647
Cleaning frequency	−16.181	4.304	−0.260	−3.759	0.000	−24.731	−7.631
Usage of a ventilation system	−16.141	6.827	−0.112	−2.364	0.020	−29.702	−2.580
People present in the room	5.824	0.825	0.355	7.063	0.000	4.186	7.162
The floor is clean and in good repair	14.523	10.620	0.125	1.368	0.175	−6.571	35.618
Cleanness of wall	−12.945	8.177	−0.113	−1.5883	0.117	−29.187	3.297
Cleanness of the window and door	3.163	6.753	0.026	0.468	0.641	−10.251	16.577
The ceiling of the room clean	−51.690	7.906	−0.422	−6.538	0.001	−67.394	−35.985

## Discussion

Poor indoor air quality in healthcare institutions may cause sick hospital syndrome including respiratory and skin symptoms. It may lead to hospital-acquired infections in patients (2). Hospital environments may be dynamic environments affected by weather conditions, visitors, and outdoor air contaminants (21).

The current study aimed to assess the indoor air bacterial load, isolates, and antimicrobial susceptibility patterns of pathogens among selected wards and staff offices at Adare General Hospital. This result showed that the indoor air bacterial load at Adare General Hospital wards and staff offices was found in the range between 210 and 3,224 CFU/m^3^. This study finding was lower than the study reported by Jimma University Hospital, which was between 2,123 and 9,733 CFU/m^3^ (22).

The possible reason for the difference was bacterial detection studies in healthcare settings are highly dependent on the study site characteristics, the detection methodologies, and the sampling used (23).

In this study, there was only one collection device and the time of air sampling from all of the selected wards and staff offices was limited (1 h for each sample). Because of the heterogeneous spread of bacteria and their different size, during air sample collection in passive sampling, some microorganisms impact on Petri dish, while others are still suspended in the air. Therefore, the longer the sampling time the more detectable the microorganisms (4). On the other hand, the bacterial concentration in the hospital environment may be affected by climate conditions, seasonal changes, and outdoor air (7).

There are no uniform international standard levels of maximum indoor air bacterial concentration, but different scholars and surveys recommend acceptable threshold limit values for indoor air bacterial load. The standard set by the World Health Organization Expert Group on the assessment of health risks of biological agents in indoor environments suggested that total indoor air bacterial load should not exceed 1,000 CFU/m^3^. If higher than this, the environment is considered as high indoor air bacterial load and causes nosocomial infection in hospital (23).

Adare General Hospital was considered an unacceptable indoor air bacterial load which was the maximum range of 3,224 CFU/m^3^ above the acceptable value that the WHO expert group suggested. Other studies indicated that the reason bacterial load was higher in hospital wards was pathogenic bacteria can be transferred from infected patients to hospital objects within the patient rooms (24). The study indicates that the indoor air bacterial loads inwards and offices at different times of day have a slight difference. Samples collected in the morning 45/50 (90.0%) were slightly more unacceptable than afternoon samples 42/50 (84%). No significant difference was observed at a *p*-value of 0.372. This is similar to the finding in Northern Ethiopia at Gondar Hospital (21) but disagrees with the finding in Adama (23). One study found a significant increase in indoor air bacterial levels in the afternoon, which may be due to higher traffic volumes and improper use of ventilation systems. These factors can cause microbes to become suspended in the air, potentially causing infections (21). The highest mean indoor air bacterial load was identified from gynecology wards with a mean of 2,542.5 CFU/m^3^, followed by pediatric ward/NICU with a mean of 2,536 and surgical ward with a mean of 2,166.8, which was similar to other previous studies reported from Gondar University Hospital (21), and the women and maternity ward of a teaching hospital in Kandy, Sri Lanka (25). Therefore, the gynecology and surgical wards showed the highest pathogenic bacteria concentrations were detected; particularly, the pediatric ward could be due to overcrowding of people, which was indicated by other studies. In the gynecology ward, the number of visitors and the number of admitted patients were high and caused a high bacterial load in this study.

A quantitative interpretation of mean indoor air bacterial load results describing air quality in Adare general hospital was evaluated based on the sanitary standards for non-industrial premises formulated by the European Commission which considers <50 CFU/m^3^ as “very low” bacterial load, 50–100 CFU/m^3^ as “low” bacterial load, 100–500 CFU/m^3^ as “intermediate” bacterial load, 500–2,000 CFU/m^3^ as “high” bacterial load, and above 2,000 CFU/m^3^ as “very high” bacterial load (26).

According to this classification, the indoor air quality of Adare General Hospital was in the range of highly contaminated with bacteria. This might be because of the high number of visitors in and out of the wards, the high number of patients, and the presence of a high number of beds in the wards during sampling time. This indicates increasing the shading of bacteria and agitation of air as was indicated in previous studies (27). This may be leading to an increment of the pathogenic organisms that cause hospital-acquired infection for patients and health workers.

The successful management of patients and staff with bacterial infection depends on the early identification of pathogens and the selection of antibiotics against the organism (6). Antibiotics are the pillar of medical care and play a major role in both prophylaxis and the treatment of infectious diseases. The issues of their availability, selection, and proper use are of critical importance to the global community (28).

In this study, findings from total air samples processed during the study period out of 100 samples demonstrated bacterial growth. This implies many pathogenic bacteria remain suspended in the hospital air during sampling time. The current finding showed that 84 (72.4%) of gram-positive and 32 (27.6%) of gram-negative bacteria from the total isolated pathogenic bacteria. which is comparable to a study carried out by Bahir Dar Felege-Hiwot Referral Hospital which reported 81.6% gram-positive, and 18.4.% of gram-negative bacteria (29).

The predominance of gram-positive organisms was reported in some other recent studies (6, 30). The possible reason for the predominant gram-positive pathogenic bacteria isolated was the inability of gram-negative bacteria to survive for long periods in the aerosolized state as was explained in other studies and the inability to resist harsh conditions like drying. Gram-positive bacterial cell wall contains a high content of peptidoglycan, which resists desiccation or dryness as moisture and soiled environments favor the growth and persistence of gram-negative bacteria (12, 21).

In the present study, *S. aureus* was 37.1% and *CoNs* were 26.7%, which were the predominant and abundant isolated pathogen*s* from gram-positive. This result was found in agreement with studies carried out in Gondar University Hospital (6), and these isolated pathogens are a known cause of hospital-acquired infection, especially among inpatient departments such as gynecology and surgical wards because of the immune-suppressed patients admitted to the hospital. This study finding showed that gram-negative pathogenic bacteria such as *E. coli* were 11.2% and pseudomonas species were 8.6%. This was in agreement with the study reported from the indoor air sample, mainly 47.18% of *Staphylococcus aureus*, CoNS, and *E. coli* were identified in the Hospital of Kathmandu District in India (31) and hospitals in Iran and Nigeria (30, 32), Sri Lanka (25), and Teaching hospital Northeast China (33).

Antimicrobial-resistant bacteria pose growing concerns. The widespread use of drugs, especially inappropriate use of antibiotics, and lack of performing antimicrobial susceptibility tests are responsible for resistance development toward antimicrobials (7). In this study, concerning antibiotic resistance profile of the isolates has reported high levels of resistance to the commonly prescribed antibiotics. This study revealed that the highest level of antibiotic resistance shown by *S. aureus* isolates was observed in 60.5% of ampicillin and 55.8% of chloramphenicol. These are locally the antibiotic of choice for the treatment of infections caused by these bacteria. This study finding was consistent with the study conducted in the Hospital of Kathmandu District in India (31), and the report from Gondar Hospital (6). Based on these data, *S. aureus's* resistance to ampicillin is slightly high in Ethiopia. Ampicillin is an available antibiotic in Ethiopia because it is prescribed for different infections.

In addition to these bacteria, CoNS have shown 48.4% resistance to chloramphenicol, tetracycline, and ciprofloxacin. This study finding is in line with a study report from Jimma (34) and Adama Hospital Ethiopia (23), but disagree with the maternity hospital in Iran (28). The variation of bacterial drug-resistant may be because of rationale antibiotic usage, which varies from hospital to hospital as suggested by other studies (8).

This study shows that Pseudomonas spp. were 60.0% resistant to cephalexin and 50.0% to both ciprofloxacin and gentamicin. This was consistent with a study reported from Hawassa University Hospital (9), a maternity hospital, in Iran (28), and an Indian hospital (31). It might be suggested that another study for resistance of bacteria was because of the mutation of genes (23).

This study revealed that *E*. coli bacteria showed 46.2% resistance to ciprofloxacin and 38.5% to penicillin. The resistance profile of isolates to individual drugs indicated that the isolated bacteria of *K*. *pneumonia* showed 77.8% resistance to cephalexin. This finding was consistent with the study conducted in Hawassa (9), Istanbul, Turkey (35), cephalexin resistance among *K*. *pneumonia* strains is slightly high because the drug cephalexin is an available antibiotic in Ethiopia and its broad-spectrum activity is prescribed for different hospital-acquired infections. The possible reason for common antibiotics could resist the isolated pathogenic bacteria was due to irrational prescription of antibiotics and their misuse by patients which were indicated by other studies (36).

In this study, the overall multidrug resistance rate of isolated bacteria was 87% resistance to more than two antimicrobial classes. The present study found a higher prevalence of multi-drug resistance (MDR) compared to previous reports from Bahir Dar Felege-Hiwot referral hospital (29), where a rate of 75% was documented, and Hawassa referral hospital (27), where a rate of 73.8% were reported. Furthermore, the MDR rate observed in this study (65.4%) was also higher than that reported in a Nigerian hospital (8). The most common reasons for the multidrug resistance of bacteria were the indiscriminate use of antibiotics without drug sensitivity testing, poor hospital hygienic conditions, and inadequate surveillance as suggested by other previous studies (9).

Several studies have indicated that biological indoor air pollutants pose potential hazards to patients and medical staff in hospitals (7, 23). It could be hypothesized that many determining related factors play a role in bacterial load increment in hospitals (7).

In the present study, there was a negative linear association between bacterial loads with the frequency of cleaning, room temperature, and proper usage of the ventilation system. Environmental factors particularly insufficient ventilation played a crucial role in the increment of bacterial load in the indoor air (21).

Similarly, in this study, there was a significant relationship between usage of the ventilation system and bacterial count. However, there was no significant difference in bacterial load between rooms with cleanness of the window and door. The results of this study suggest that reducing microbial contamination in hospital rooms depends on the use of windows rather than their cleanliness. In particular, the air exchange made possible by the use of windows has been proven to effectively reduce bacterial contamination.

### Limitations of the study

- One of the limitations of this study was the bacterial load of indoor air quality represents only the condition during sampling time and it may not represent indoor air bacterial load during other times (temporal association not clear).
- This study may have limitations on the identification of bacterial isolation with limited methods, which were based only on the morphological characteristics of bacterial culture (colonies). Due to this, only dominant bacteria were isolated or unusual morphological structure bacteria were not isolated as compared with DNA-based molecular method.
- The effectiveness of data collection through passive air sampling can be influenced by environmental parameters due to the inherent characteristics of the technique used. In particular, reverse diffusion can potentially lead to an under- or overestimation of the bacterial load.
- This study may have limitations on the transmission dynamics of bacterial strains and antibiotic-resistant genes within a hospital environment due to limited methods.

## Conclusion

In the present study, the degree of indoor air bacterial loads was far beyond the acceptable limits at Adare General Hospital as compared with the standards.

The highest mean indoor bacterial load was identified from different departments of the hospital which were the inpatient department, outpatient department, and staff office, respectively, and the predominant pathogenic bacteria were *S. aureus, CoNS, Pseudomonas* spp*., E.coli*, and *K. pneumonia* since those were the common known cause of nosocomial or hospital-acquired infection.

This study indicated the alarmingly high levels of antibiotic resistance for prescribed drugs. The higher levels of multidrug resistance were observed among gram-positive pathogenic bacteria, *S. aureus*, and *CoNS* were highly resistant to ampicillin, chloramphenicol, and ciprofloxacin. Therefore, antimicrobial resistance is a growing global problem.

The main influencing factors that contributed to the high range of bacterial load were low cleaning frequency, the temperature of the room, and usage of the ventilation system.

## Recommendations

Based on the findings of the study, the following recommendations are forwarded:

⊳ Hawassa city administration health department and the hospital need attention to the air quality, which is important in the prevention of nosocomial infections.
⊳ Improve the cleanness of hospital rooms, especially by increasing cleaning frequency, proper use of ventilation, and restricting the number of visitors in the wards to minimize indoor air bacterial loads.
⊳ In addition, attention should be given to controlling physical factors such as temperature which favor the growth and multiplication of pathogenic bacteria in indoor environments of the hospital.
⊳ Adare general hospital administrators should work in collaboration with other hospitals.
⊳ Multiple drug resistance of isolates to antimicrobials was alarmingly high. Therefore, any empirical prophylaxis and treatment need a careful selection of effective drugs.

## Data availability statement

The raw data supporting the conclusions of this article will be made available by the authors, without undue reservation.

## Ethics statement

The ethical clearance was obtained through the Research and Ethics Clearance Committee (Research Review Board) of the Hawassa University College of Medicine and Health Sciences with Ref. No: IRB/172/13 before data collection. The latter was obtained by the Department of Environmental Health and entrusted to the administration of the Adare General Hospital. A letter of cooperation was drawn up by Adare General Hospital (AGH) and submitted to the appropriate departments of this hospital. Data was collected and information obtained during this study was kept confidential and was used only for this study.

## Author contributions

NG: Data curation, Methodology, Supervision, Conceptualization, Formal analysis, Investigation, Resources. The final manuscript was prepared by YA. BB has contributed equally for this manuscript preparation, like Data curation, Conceptualization, Formal analysis, Writing—review & editing, Software, and Validation. All authors actively participated during the conception of the research issue, development of the research proposal, sample collection, analysis and interpretation, and writing of various parts of the research report. All authors read and approved the final manuscript.
